# Potential of endophytic *Beauveria bassiana* against *Coraebus* (Coleoptera: Buprestidae) oak borers

**DOI:** 10.1002/ps.70473

**Published:** 2025-12-30

**Authors:** Walaa Morda, Alessia Vinci, Andrea Lentini, Roberto Mannu, Maurizio Olivieri, Luca Ruiu

**Affiliations:** ^1^ Department of Agricultural Sciences University of Sassari Sassari Italy

**Keywords:** *Quercus suber*, *Coraebus florentinus*, *Coraebus undatus*, entomopathogenic fungi, endophytism

## Abstract

**BACKGROUND:**

Oak borers in the genus *Coraebus*, including the bark‐ and the wood‐boring beetles *C. florentinus* and *C. undatus*, are major pests of the cork oak tree *Quercus suber*, and when their population densities are high, effective forest protection measures become critical. The endophagous behaviour of *Coraebus* species and the limited understanding of their biology, hamper the development of timely and effective management strategies.

**RESULTS:**

The novel strain UNISS22 of the entomopathogenic fungus *Beauveria bassiana*, isolated from the forest ecosystem, demonstrated strong insecticidal potential against the model coleopteran *Tenebrio molitor* and the two target *Coraebus* species, achieving up to 100% mortality in both larvae and adults, with efficacy shown to be concentration‐dependent. *Beauveria bassiana* UNISS22 was found to exhibit endophytic behaviour in *Quercus* plants and to possess a distinctive ability to produce increased fungal biomass. Genomic analyses revealed a set of genes encoding proteins related to the insecticidal potential, including genes involved in adhesion to the host (adhesins and hydrophobins), in penetration and infection (chitinases, proteases and subtilases), and in the synthesis of bioactive secondary metabolites. Gene sequence analyses revealed a significant level of divergence in strain UNISS22, supporting the presence of distinct biological properties and functional potential compared with other *B. bassiana* strains.

**CONCLUSION:**

The biological properties and insecticidal potential of *B. bassiana* strain UNISS22 provide valuable insights for developing eco‐friendly, integrated management strategies to protect forests from *Coraebus* beetle infestations. © 2025 The Author(s). *Pest Management Science* published by John Wiley & Sons Ltd on behalf of Society of Chemical Industry.

## Graphical abstract

This study demonstrates that an endophytic, oak‐associated strain of *Beauveria bassiana* exhibits adaptations to *Quercus* plants and potential against *Coraebus* boring beetles, supported by distinctive biological traits.

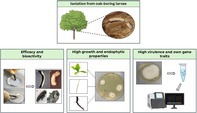

## 1 INTRODUCTION

Oak borers of the genus *Coraebus* (Coleoptera: Buprestidae), in particular the bark‐ and the wood‐boring beetles *Coraebus florentinus* Herbst and *Coraebus undatus* Fabricius, are major pests of the cork oak tree *Quercus suber* L., with a significant impact on the economy of the cork industry and the health of forest ecosystems in different geographical areas.^1^, ^2^ The activity of these species contributes to the decline of forest ecosystems across Europe,^3^ due to their boring activity and their association with microbial plant pathogens, for which they can serve as vectors.^4^ Although they share a borer lifestyle, these two species cause different types of damage and feed on distinct parts of the plant tissues. More specifically, *C. florentinus* larvae typically feed by boring longitudinal tunnels under the bark of young, vigorous branches. As the larva develops, its activity disrupts sap flow, often leading to death of the corresponding branch, typically coinciding with the insect metamorphosis.^5^ By contrast, young larvae of *C. undatus* feed in the phellogen layer by boring typical sinuous tunnels with an elliptical cross‐section, which later become blackish. Older larvae pierce the cork to prepare the pupation chamber, after which the adults emerge, usually in late spring/early summer. All this damage to plant tissues impairs the regenerative ability of the tree and reduces its production of acorns, cork and wood.^6^, ^7^ In addition, it reduces the quality and commercial value at hulling, making the removal process more difficult.^8^ Consequently, *C. undatus* receives special attention because of its economic impact, which can be particularly significant under conditions of heavy infestation.^3^, ^9^

The internal feeding behaviour of *Coraebus* species, along with a limited understanding of their biology, hampers the development of timely and effective management strategies. The lack of commercially approved active substances that specifically target these pests and are compatible with the forest environment adds further issues.^10^ Accordingly, there is an urgent need to develop new and alternative management approaches that are compatible with the forest ecosystem. One frontier in this direction is the use of naturally occurring microbial control agents that are effective in their natural containment. These include entomopathogenic fungi such as *Beauveria bassiana* (Balsamo‐Crivelli) Vuillemin (Ascomycota: Hypocreales). This entomopathogen has been successfully used in agriculture and typically acts by contact through the adhesion of its conidia to the surface of the target insect body, where it germinates under favourable conditions. This triggers a mechanical and enzymatic action that results in the tegumental barrier being overcome by penetrating hyphae. The fungus reaches the internal tissues where the pathogenic process induces ultrastructural, biochemical and physiological changes that, together with a toxic action, cause the death of the insect.^11^

A key step in the pathogenic process is therefore the initial physical interaction of the fungus with the host, which is more difficult in the case of endophagous larvae. Nevertheless, endophytic behaviour has been observed in several *B. bassiana* strains, which are capable of colonizing internal plant tissues without causing damage to the plant. This internal presence may enable the fungus to infect endophagous larvae, suggesting its potential use in the biological control of insect borers.^12^ To date, no reports are available on the potential of endophytic *B. bassiana* strains against *Coraebus* boring beetles.

The main hypothesis of this study is that an entomopathogenic fungus, naturally adapted to endophytic behaviour and capable of acting against *Coraebus* species, may be a promising tool for *Coraebus* management. Accordingly, a new strain of *B. bassiana*, isolated from a *Coraebus* larva during an infestation in an oak forest, was tested against *C. florentinus* and *C. undatus* for its insecticidal potential, including a comparison with currently available commercial strains. In addition, observations were made on its endophytic behaviour. Finally, the whole genome of the new strain was sequenced and annotated, revealing a gene profile with specific adaptations to insecticidal action and endophytism.

## 2 MATERIALS AND METHODS

### 2.1 *Beauveria bassiana* strain isolation

**Figure 1:**
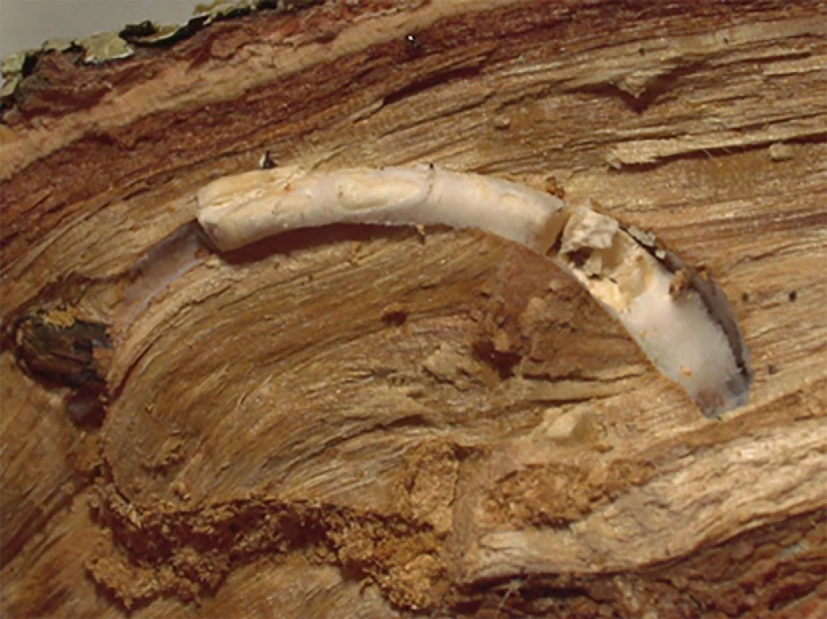
*Coraebus florentinus* larva covered with *Beauveria bassiana* mycelium inside a gallery bored into an oak branch, carefully sectioned for observation.

*Beauveria bassiana* strain UNISS22 was isolated from a naturally infected *C. florentinus* larva found inside a branch of an infested oak tree in Sardinia (Italy) during forest surveys in spring 2022. The endophagous larva was dead and completely covered with a compact white fungal mycelium (Fig. 1). The dead endophagous larva was surface‐sterilized with ethanol (70%) and sodium hypochlorite (0.05%), washed with sterile water,^13^ to eliminate external contaminants, and then placed on potato dextrose agar (PDA) supplemented with 0.05% streptomycin, incubated at 25 °C in the dark to allow the internal fungus to grow in isolation. Monosporic cultures were obtained to ensure isolate purity for morphological observations and preliminary identification according to Humber,^14^ followed by whole‐genome sequencing for definitive species confirmation, as described below. The strain was cryo‐preserved at −80 °C in cryovials containing a glycerol solution at the Microbial Collection of the University of Sassari.

Other *B. bassiana* strains used as references in this study were ATCC74040, GHA and PPRI 5339, which represent the active substances of the commercial products Naturalis (Biogard, Grassobbio, Italy), Botanigard (Certis Belchim B.V., Utrecht, Netherlands) and Velifer (BASF SE, Ludwigshafen, Germany), respectively.

### 2.2 Fungal growth conditions and preparations

*Beauveria bassiana* was routinely grown on PDA plates at 25 °C to allow good production of mycelium and aerial conidia. For bioassays, conidia were harvested from 15‐day‐old cultures, because this incubation period—based on viability experiments conducted to standardize procedures—ensured a conidial viability greater than 90%. Conidia were collected by gently scraping the colony surface with a glass spatula using a sterile solution (10 mL) of water and 0.02% Tween 80. When necessary, conidial suspensions were filtered to remove hyphal fragments and other cultural debris. Conidial suspensions were quantified using a Neubauer chamber, and the concentration was adjusted as required for bioassays.

### 2.3 Comparative fungal growth observations

The growth of *B. bassiana* strain UNISS22 was monitored after inoculation on PDA in comparison with the reference strains ATCC74040, GHA and PPRI 5339. To monitor horizontal growth on plates, a hole (8 mm in diameter) was made in the centre of a Petri dish (9 cm in diameter) and inoculated with 100 μL of a fresh fungal suspension (10^7^ conidia/mL). Vertical growth was measured instead in 50‐mL tubes containing PDA in which the whole medium surface was inoculated with 100 μL of the same fungal suspension. Cultures were then incubated in the dark at 25 °C, and their horizontal and vertical growth was measured with a millimetre ruler daily during the following 12 days. At least three measurements were taken in different directions on each mycelial body in the plate to determine the average diameter, so that the shape of its surface was approximately circular. Measurement data were based on three replicates, and all experiments were repeated four times. Measurements of horizontal and vertical growth were combined to calculate the volume of fungal biomass produced in culture (base area × height of mycelial mass). Comparisons were made based on measurements taken 15 days after initial inoculation. Measuring both horizontal growth (colony expansion on the PDA surface) and vertical growth (mycelial height) allowed us to estimate the three‐dimensional volume of fungal biomass. This volumetric approach provides a more accurate and comprehensive assessment of fungal growth and biomass production than surface measurements alone, enabling meaningful comparisons among different *B. bassiana* strains.

### 2.4 Insect collection and maintenance

The coleoptera used in this study were the two target species, *Coraebus florentinus* and *C. undatus*, and the lab‐reared model coleopteran *Tenebrio molitor* L.

Fifth‐instar larvae of *C. florentinus* were collected from cork oak branches in heavily infested forests in Sardinia (Italy), in the area of Buddusò (north‐east of the island), and directly used in bioassays, after a short acclimatization period (24 h) in the laboratory at 25 °C. *Coraebus undatus* adults were collected in the cork oak forests of Tempio Pausania (north‐east Sardinia) using emergence traps. This approach was chosen because of the difficulty in rearing these species through their entire life cycle in the laboratory.

*Tenebrio molitor* adults, pupae, and larvae used in the bioassays were routinely provided by the laboratory rearing facilities of the Department of Agricultural Sciences of the University of Sassari where this species was reared on wheat bran as substrate and diet, supplemented with moisture sources such as small pieces of fruit and vegetable residues.

### 2.5 Insect bioassays

Bioassays were conducted by exposing different stages of *C. florentinus*, *C. undatus* and *T. molitor* to *B. bassiana* UNISS22 conidial suspensions, in order to determine the lethal effects and the ability of the fungus to complete its pathogenic cycle on the host. In all cases, insects (larvae, pupae or adults) of the different coleopteran species were individually immersed in a suspension of conidia (5 mL) or in distilled water containing the same concentration of Tween 80 as used in the fungal suspensions (control) for 30 s before being maintained together in a group of ten individuals in a Petri dish (9 cm in diameter). Before this treatment, field‐collected *Coraebus* larvae were surface‐sterilized with 70% ethanol for 1 min, followed by treatment with 0.05% sodium hypochlorite for 1 min. The larvae were then rinsed twice with sterile water to remove external microorganisms and minimize interference from contaminating microbes, ensuring that the observed effects were attributable to the applied *B. bassiana* treatment. Petri dishes, which represented the experimental unit, were incubated in the dark at 25 °C for observations over the following days. Different conidia concentrations were tested on *T. molitor* (10^7^, 10^6^ and 10^5^ conidia/mL), whereas *Coraebus* samples were exposed to a standardized concentration of 10^7^ conidia/mL. Using *T. molitor* as a model coleopteran species allowed for multiple observations across different concentrations because of the abundant availability of laboratory‐reared individuals, thereby compensating for the limited availability of *Coraebus* specimens collected from the field.

In all cases, the experimental design involved four replicates per treatment, and each experiment was repeated two or three times over time. Plates were inspected daily to record mortality for 5 days after treatment, which proved to be a sufficient and standard period to highlight lethal effects and to allow comparison between different developmental stages. Further observations of the insects on the plates were carried out over a 2‐week‐period to monitor the development of pathological symptoms. Dead larvae were surface‐sterilized prior to plating on PDA to confirm that fungal growth originated from internal infection rather than external contaminants. Thus, the mortality refers to death caused by *B. bassiana* infection.

According to the same experimental design, comparative mortality experiments were conducted on *T. molitor* larvae with *B. bassiana* UNISS22 and reference strains ATCC74040, GHA and PPRI 5339, with the purpose of assessing possible differences in virulence between strains when the model host used in this study was exposed to their conidia.

### 2.6 Endophytic behaviour observation

The ability of *B. bassiana* strain UNISS22 to penetrate plant tissues and translocate within the plant was investigated by applying conidial suspensions at different concentrations to oak tree seedlings and then examining internal tissues for the presence of the fungus. For this purpose, the roots of 1‐year‐old (30 cm tall) cork oak tree seedlings were washed, surface‐sterilized using 70% ethanol for 1 min, followed by treatment with 0.05% sodium hypochlorite for 2 min, then rinsed in distilled water and immersed in conidial suspensions or just water containing the same concentration of Tween 80 as used in the fungal suspensions (control) for 30 min. Plants were then transplanted into pots with soil, kept in good conditions in a room at 25 °C with natural photoperiod (14:10 h light/dark). The experimental design involved three replicates of four plants each, and different treatments (10^7^ and 10^6^ conidia/mL, and untreated control). After 3 weeks, five small portions (3 mm in diameter) of roots, stems and leaves were excised from each plant and surface‐sterilized before being placed on PDA plates supplemented with 0.05% streptomycin to allow growth of any fungal mycelium or propagules present in the internal plant tissues. Plates were incubated at 25 °C, checked daily for the presence of *B. bassiana* white mycelium, and then sampled to confirm identification.

### 2.7 Genomic analysis on *B. bassiana* UNISS22

For genomic analysis, DNA from *B. bassiana* UNISS22 was extracted from a pure culture using the Fungi/Yeast Genomic DNA Isolation Kits (catalogue number 27300; Norgen Biotek Corp, Canada) according to the manufacturers’ instructions.

The DNA libraries were prepared using an Illumina DNA prep kit (Illumina, San Diego, CA, USA) with unique dual indexes and sequenced on an Illumina NovaSeq 6000 platform at BMR Genomics (Padua, Italy) in 150PE format following the manufacturer's instructions. The quality of raw paired‐end (PE) reads was checked using FASTQC v.0.11.8.^15^ MetaPhlan v.4.0.1^16^ was used for microbial profiling to assess sample content, including potential contaminants. SPAdes v.3.15.5^17^ was used for *de novo* assembly, whereas QUAST v.5.2.0^18^ and BUSCO v.5.4.3^19^ were used to evaluate assembly quality.

General gene prediction and annotation were preliminarily done with eggnog‐mapper v.2.1.9 on eggNOG DB database v.5.0.2.^20^ The genome was then analysed for the presence of genes potentially involved in entomopathogenic activity or related to the endophytic properties of the fungus. These genes were selected according to available literature data and also by consulting the Pathogen–Host Interactions database (PHI‐base).^21^ The sequence of these genes was aligned with the sequence of the reference genome strain ARSEF 2860 (accession number: GCF_000280675.1) to determine the degree of homology, using the BLAST+ v.2.16.0 suite of NCBI.^22^ Further sequence comparisons were made with other *B. bassiana* genomes available on NCBI, including strain ATCC74040 used in this study.

### 2.8 Statistical analysis

Data processing and statistical analyses were performed using R software, v.4.4.3.^23^ One‐way analysis of variance (ANOVA) followed by Tukey's post‐hoc test^24^ was used to analyse fungal growth data, whereas two‐way ANOVA (factors: conidia concentration and plant portion) followed by Tukey's post‐hoc test was used to analyse fungal endophytic behaviour. Mortality data were analysed using Firth's bias‐reduced logistic regression to account for complete separation in some groups (e.g. 100% mortality).^25^ A Generalized Linear Mixed Model (GLMM) with a binomial error distribution and logit link was used to analyse the effect of treatment and time on survival.^26^ Post‐hoc pairwise comparisons among treatments at each time point were performed using estimated marginal means, with Tukey adjustment for multiple testing. Time to death means were compared between two different insect species exposed to *B. bassiana* using *t*‐tests.

## 3 RESULTS

### 3.1 Fungal growth behaviour

Growth indicators of the strain UNISS22 of *B. bassiana* 2 weeks after inoculation on PDA in comparison with other strains are shown in Table 1. Although strain ATCC74040 showed the greatest horizontal growth (*F*_3,12_ = 6.22; *P* = 0.00856), strain UNISS22 revealed the greatest ability to grow vertically (*F*_3,12_ = 5.462; *P* = 0.0134). By combining horizontal colony expansion and vertical mycelial height measurements, we estimated the three‐dimensional biomass volume produced by each strain. Using this volumetric approach, strains UNISS22 and ATCC74040 demonstrated comparable biomass production, which was approximately double that of strains GHA and PPRI 5339 (*F*_3,12_ = 33.95; *P* < 0.0001).

**Table 1:** Comparative production of *Beauveria bassiana* fungal biomass on potato dextrose agar (PDA)

*B. bassiana* strain	Horizontal growth (mm)	Vertical growth (mm)	Biomass volume (mm^3^)
ATCC74040	34.5 ± 1.1 a	5.8 ± 0.5 a	7074.7 ± 294.6 a
GHA	27.2 ± 5.4 b	4.8 ± 1.0 a	3487.0 ± 585.2 b
PPRI 5339	24.9 ± 3.9 b	6.0 ± 0.8 a	3666.5 ± 1124.3 b
UNISS22	26.2 ± 1.3 b	9.8 ± 3.5 b	6737.9 ± 247.2 a

Different letters within a column indicate significantly different means (one‐way analysis of variance, followed by Tukey's test, *P* < 0.05).

### 3.2 Insect bioassays

**Figure 2:**
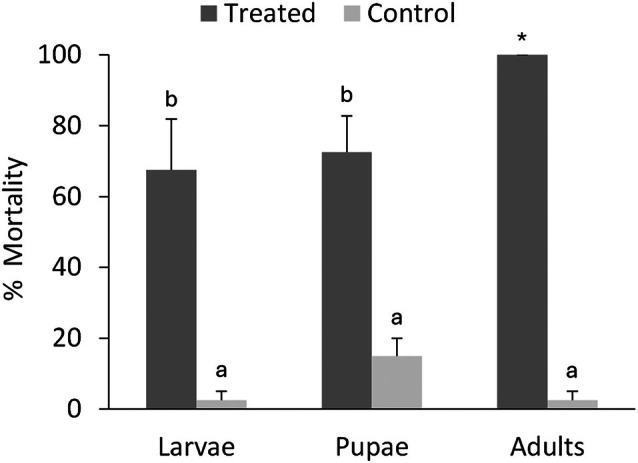
Estimated mortality (mean ± SE) for different *Tenebrio molitor* stages exposed to *Beauveria bassiana* UNISS22. Different letters above bars indicate significantly different mortality rates based on Firth logistic regression of raw mortality counts (dead *versus* alive) with Tukey‐adjusted comparisons (*α* = 0.05). Asterisks (*) mark groups with complete (100%) mortality across all replicates.

Different stages of *T*. *molitor*, used as a model insect, showed varying susceptibility when exposed by contact to *B. bassiana* UNISS22 suspensions at an average concentration of 10^7^ conidia/mL. Mortality exceeded 70% in larvae and pupae, and reached 100% in adults, 5 days after treatment, whereas control groups showed low mortality (2.5–15%) (Fig. 2). Firth logistic regression showed a highly significant effect of treatment on insect mortality (*χ* ^2^ = ∞, *P* < 0.001), with treated insects experiencing substantially higher mortality than controls. The interaction between treatment and stage was also significant for both larvae (*χ* ^2^ = 4.60, *P* = 0.032) and pupae (*χ* ^2^ = 15.83, *P* < 0.001), indicating that the effect of treatment varied among developmental stages. Stage alone was not significant for larvae (*P* = 1.00), and marginally non‐significant for pupae (*P* = 0.054). The overall model was highly significant (likelihood ratio test = 169.24, df = 5, *P* < 0.001; *n* = 240).

**Figure 3:**
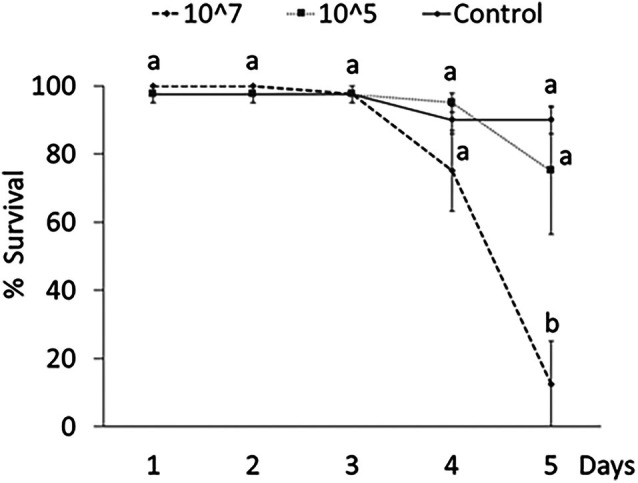
Over time survival (mean ± SE) of *Tenebrio molitor* adults exposed to different concentrations (conidia/mL) of *Beauveria bassiana* UNISS22. Different letters indicate significantly different means (Generalized Linear Mixed Model binomial, Tukey‐adjusted; *P* < 0.05).

**Figure 4:**
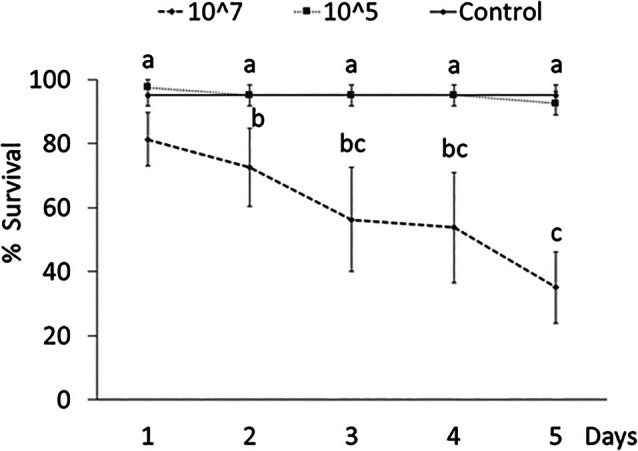
Over time survival (mean ± SE) of *Tenebrio molitor* larvae exposed to different concentrations (conidia/mL) of *Beauveria bassiana* UNISS22. Different letters indicate significantly different means (Generalized Linear Mixed Model binomial, Tukey‐adjusted; *P* < 0.05).

Over time survival rate was significantly affected by treatment and time according to successfully converged GLMMs in *T. molitor* adults [Akaike information criterion (AIC) = 142.8; log likelihood (logLik) = −55.4] and larvae (AIC = 263.9; logLik = −115.9). A significant reduction in survival rate after 5 days was observed on both adults (*Z* = 4.25, *P* < 0.001) and larvae (*Z* = 3.89, *P* < 0.001) exposed to a higher fungal concentration (10^7^ conidia/mL) compared with control (Figs 3 and 4).

**Figure 5:**
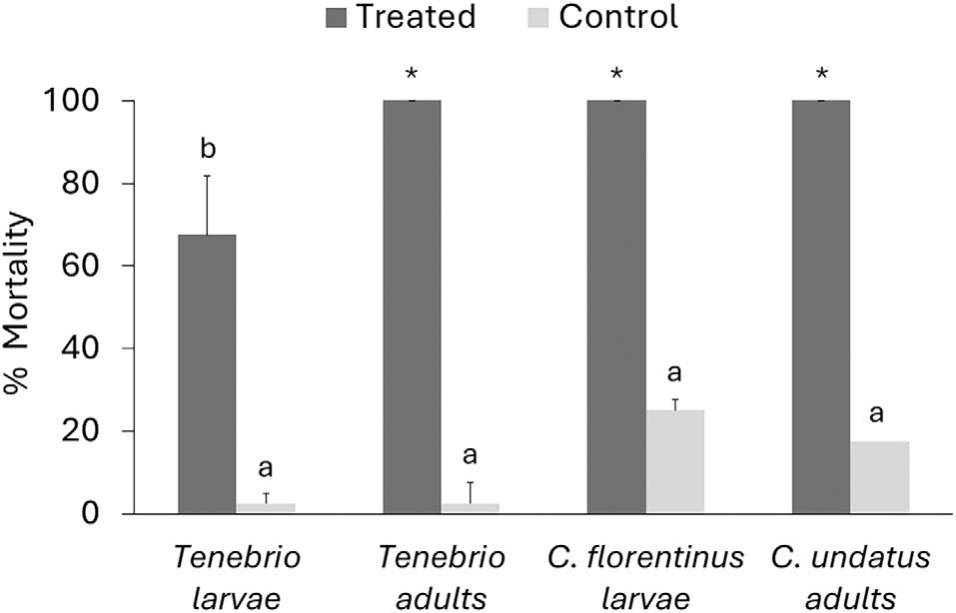
Estimated mortality (mean ± SE) for different *Coraebus* species and stages exposed to *Beauveria bassiana* UNISS22 in comparison with *Tenebrio molitor*. Different letters above bars indicate significantly different mortality rates based on Firth logistic regression of raw mortality counts (dead *versus* alive) with Tukey‐adjusted comparisons (*α* = 0.05). Asterisks (*) mark groups with complete (100%) mortality across all replicates.

*Coraebus* samples were also highly susceptible to *B. bassiana* UNISS22, with 100% mortality achieved in 5 days after treatment for both *C. florentinus* larvae and *C. undatus* adults (Fig. 5). Firth logistic regression revealed a strong effect of treatment on mortality (*χ* ^2^ = 56.70, *P* < 0.001), with significantly higher mortality observed in treated insects. The insect target also significantly affected mortality, with *T. molitor* larvae showing reduced susceptibility compared with other targets (*χ* ^2^ = 8.75, *P* = 0.003). No significant interaction was found between treatment and insect target (*P* > 0.3 for all interactions), indicating that the effect of treatment was generally consistent across stages. The overall model fit was highly significant (likelihood ratio test: *χ* ^2^ = 274.70, df = 7, *P* < 0.001; *n* = 320). Mortality results were comparable with those observed in *T. molitor* larvae and adults, also taking into account a slightly higher larval mortality in the *Coraebus* control under laboratory conditions.

The average time to death (mean ± SE) was also slightly shorter for *Coraebus* (2.58 ± 0.57 days) compared with *Tenebrio* (3.46 ± 0.39 days) larvae, although these differences were not significant (*t* = 2.0930, df = 19, *P* = 0.2188).

**Figure 6:**
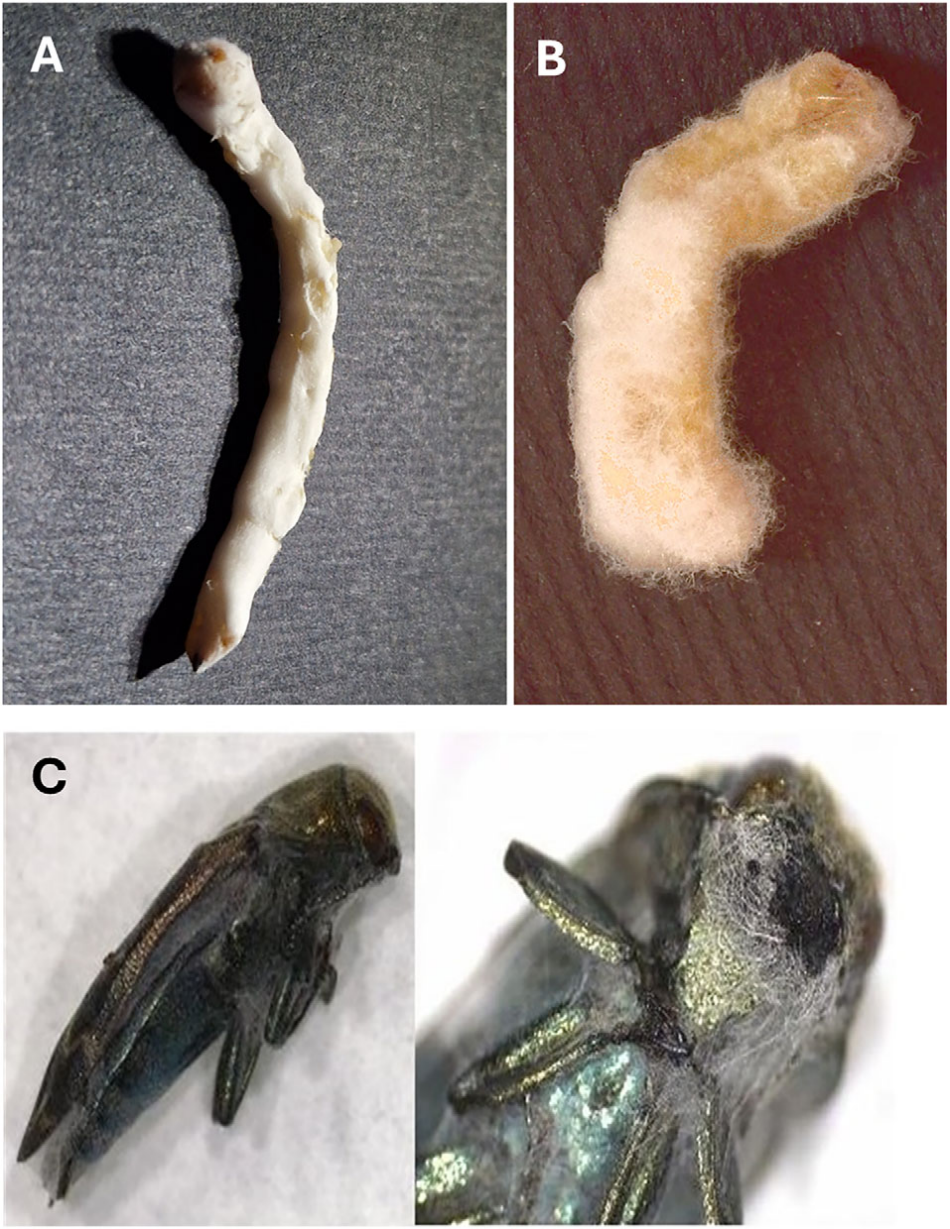
*Coarebus florentinus* larva (A) and pupa (B), and *Coraebus undatus* adult (C) infected with *Beauveria bassiana* UNISS22 and covered with fungal mycelium, conidiophores, and aerial conidia.

*Beauveria bassiana* UNISS22 showed the ability to complete the whole life cycle on different *Coraebus* host stages, culminating in the external formation of mycelium and conidiophores that produced new aerial conidia for dispersal (Fig. 6).

When *T. molitor* larvae were exposed to conidia (10^7^ conidia/mL) of different *B. bassiana* strains (UNISS22, ATCC74040, GHA and PPRI 5339), all strains were effective (average mortality >90%) compared with the control (*χ* ^2^ = 9.82, *P* = 0.044), with no significant differences in virulence between strains assessed 5 days after larval exposure (*P* > 0.05).

### 3.3 Endophytic behaviour

**Figure 7:**
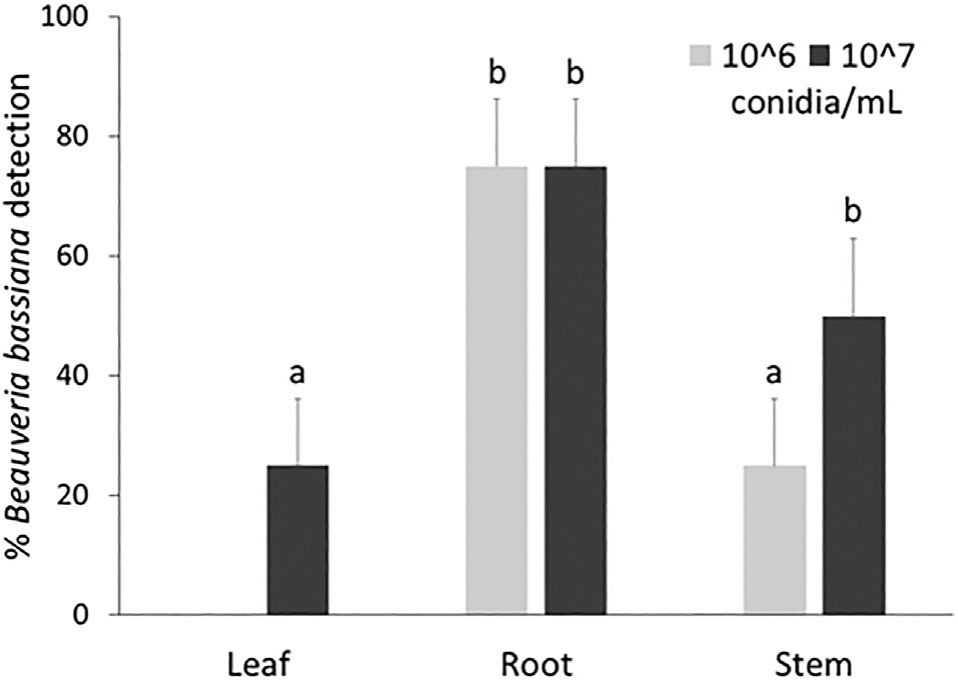
Percentages (mean ± SE) of *Beauveria bassiana* detection in different tissue samples (leaf, root, stem) of *Quercus suber* plants treated with conidia of the fungus by root immersion. Different letters above bars indicate significantly different means (two‐way analysis of variance, followed by Tukey's test, *P* < 0.05).

*Beauveria bassiana* strain UNISS22 was found inside all *Quercus suber* plants whose roots had been treated by immersion in a conidial suspension. The fungus was found most frequently in tissue samples taken from the roots, followed by the stem and leaves. A higher percentage of occurrence was associated with a higher concentration of root treatment (Fig. 7). Significant differences were therefore influenced by treatment concentration (*F*_2,31_ = 5.229; *P* = 0.011) and the portion of the plant sampled (*F*_2,31_ = 3.548; *P* = 0.041).

### 3.4 Genomic insights on *Beauveria bassiana* UNISS22

Whole‐genome sequencing resulted in a total of 61 409 652 PE reads, and the assembled genome consisted of 137 contigs (≥1000 bp), with 12 L50 contigs and an N50 value of 968 729 bp. The guanine + cytosine (GC) content was 50.90% and the total estimated size of the genome was 34 177 820 bp. Functional genome annotation resulted in the identification of several protein‐coding genes implicated in the entomopathogenic properties of strain UNISS22. These included genes related to the initial phase of conidia adhesion to host surface (adhesin and hydrophobins), to the subsequent fungal penetration into the body (chitinases and proteases), to the infection and degradation of host tissues (subtilase and other proteases), and to the production of bioactive secondary metabolites (cytochrome P450, beauvericin and tenellin synthases). Additional genes associated with the interaction of the fungus with plant tissues, such as those encoding cellulases, were also identified. Alignment of these gene sequences with the genome of the entomopathogenic reference strain ARSEF 2860 revealed that strain UNISS22 is notably distinct, showing an average sequence homology of 85–95% for these genes (Table 2). Additional comparisons with the available genome of strain ATCC74040 used in this study indicated further significant gene sequence differences (Supporting Information, Table S1).

**Table 2:** Homology of *Beauveria bassiana* UNISS22 gene selection involved in pathogenesis and endophytism

*Beauveria bassiana* strain UNISS22		Main protein functions related to virulence, pathogenesis and host–plant interaction	Homology (%) with reference strain ARSEF 2860	
Gene	Accession nos		Sequence coverage	Sequence identity
Hydrophobin	PV710265	Adhesion to the host; immune evasion	100	86.73
Hydrophobin 2	PV710266	Adhesion to the host; immune evasion	94	79.86
Adhesin protein Mad1	PV710267	Adhesion to the host cuticle	100	87.96
Chitinase 18‐3	PV710268	Cuticle degradation; internal colonization	100	84.39
Chitinase 18‐4 (Chi‐6)	PV710269	Cuticle degradation; internal colonization	100	95.79
Class V chitinase Chi100	PV710270	Cuticle degradation; internal colonization	100	93.36
Subtilase‐like protein	PV710271	Cuticle degradation; penetration	100	91.28
Subtilisin‐like protease PR1G	PV710272	Cuticle degradation; penetration	100	91.94
Alkaline serine protease AorO	PV788230	Breaking down structural proteins	100	93.00
Glucan‐1,3‐beta‐glucosidase	PV710279	Fungal development and host infection	100	89.63
Beta‐1,6‐glucanase precursor	PV710280	Fungal development and host infection	100	94.69
Cytochrome P450 CYP65T7	PV710273	Secondary metabolite biosynthesis	100	92.84
Cytochrome P450 CYP65A1	PV710274	Secondary metabolite biosynthesis	100	94.64
Cytochrome P450 CYP623C1	PV710275	Secondary metabolite biosynthesis	100	89.30
Beauvericin biosynthetic protein	PV788231	Core biosynthetic protein for beauvericin	96	89.58
Tenellin polyketide synthase	PV788232	Key enzyme for tenellin biosynthesis	100	86.51
Cellulase‐like‐protein	PV710276	Cellulose degradation	100	91.16
Cellulase	PV710277	Cellulose degradation	100	93.71
Beta‐glucosidase‐6	PV710278	Cellulose degradation	100	95.18

## 4 DISCUSSION

*Beauveria bassiana* strain UNISS22, isolated from a naturally infected *Coraebus florentinus* larva found inside an infested oak branch, proved to be an effective entomopathogen of coleopterans, as determined in laboratory bioassays using the model species *Tenebrio molitor*. These experiments have also documented a concentration‐dependent insecticidal effect and the ability of the fungus to attack all stages (larvae, pupae, adults) of the insect and complete the pathogenic process. Similarly, *B. bassiana* strain UNISS22 was able to kill larvae and adults of different *Coraebus* species by carrying out its entire biological cycle on the host, up to the external production of mycelium and conidiophores bearing new aerial conidia for dispersal.^27^ This is the first report of larval‐borne *B. bassiana* entomopathogenic efficacy against *Coraebus* species. These findings corroborate several studies reporting the pathogenicity of *B. bassiana* administered at comparable concentrations to different boring beetle species, including the spruce bark beetle *Ips typographus* L. (Coleoptera: Curculionidae),^28^ the elm bark beetle *Scolytus scolytus* Fabricius (Coleoptera: Scolitidae),^29^ the red turpentine beetle *Dendroctonus valens* LeConte (Coleoptera: Curculionidae: Scolytinae) and *C. florentinus*.^30^ However, many promising results have come from laboratory studies in which the target insects were exposed by direct contact with the fungus, whereas real field conditions require the fungus to be able to reach the larvae within the tunnels dug in the wood or bark.^13^ Accordingly, we conducted observations on the growth behaviour of the fungus and its ability to penetrate plant tissues. Comparative experiments with other commercially available strains revealed that *B. bassiana* strain UNISS22 has pronounced biomass‐producing capabilities, which may be related to greater pathogenicity and diffusion capacity across environmental surfaces such as soil and plant material, thereby enhancing fungal persistence. However, it would be more relevant to determine whether such differences in behaviour could lead to an increased capacity of the fungus to grow on infected larvae. Equally important is the ability of the fungus to colonize the plant, particularly in the context of managing endophagous pest species through endophytic activity.^11^ *B. bassiana* UNISS22 showed significant endophytic capacity, penetrating tissues and spreading internally via the vascular system, corroborating patterns observed in other strains of this entomopathogenic species.^31^ Consistently, *B. bassiana* UNISS22 was detected not only in the roots, but also in the stems and leaves of oak plants treated via root immersion. Endophytic colonization by certain *B. bassiana* strains is known to interact with plant physiology by promoting growth, inducing systemic resistance to phytopathogens, and enhancing tolerance to abiotic stresses.^32^, ^33^ Additional effects against insect herbivores may be associated with the broad fungal bioinsecticidal properties.^12^ Because these effects are often strain‐specific, they will need to be specifically investigated for *B. bassiana* UNISS 22. An experimental approach could involve applying the *B. bassiana* strain using a wound dressing methodology, as successfully reported against the bark and wood borer olive pest *Euzophera pinguis* Haworth (Lepidoptera: Pyralidae).^34^

Analysis of the genome of *B. bassiana* strain UNISS22 revealed a diverse set of genes encoding proteins related to insecticidal potential. These included genes involved in the early stages of conidial adhesion to the host surface, such as those encoding adhesins and hydrophobins.^35^, ^36^ Other genes were linked to subsequent fungal penetration into the host body, including those encoding chitinases and proteases.^37^, ^38^ Genes responsible for infecting and breaking down host tissues, such as subtilases and additional proteases, were also identified,^39^ along with those involved in the synthesis of bioactive secondary metabolites like cytochrome P450 enzymes, beauvericin and tenellin synthases.^40^, ^41^ In addition, genes related to interactions with plant tissues, such as those encoding cellulases, were found.^13^ Interestingly, the sequences of these genes showed significant variations compared with their homologues in the reference genome of *B. bassiana* strain ARSEF 2860, as well as in other entomopathogenic strains, including *B. bassiana* strain ATCC74040, which demonstrated comparable efficacy against the model species *T. molitor* in our experiments.^42^ However, in previous histological studies, leaves inoculated with this strain showed fungal activity restricted to the application site, with no spread into surrounding tissues following germination.^43^, ^44^ Conversely, strain UNISS22 was detected in plant tissues beyond the initial application site, confirming its ability to colonize endophytically and spread systemically. This finding supports its potential use against *Coraebus* pests, in line with similar approaches using specific *B. bassiana* strains targeting other pest species.^45^

Taken together, these findings suggest that strain UNISS22 may possess unique characteristics and potential related to *Coraebus*, the host from which it was originally isolated. Comparative analysis of gene sequences implicated in pathogenicity and endophytism revealed a significant level of divergence between UNISS22 and other *B. bassiana* strains, indicating the presence of distinct genetic features that may underlie its unique biological properties and functional capabilities. Moreover, the presence of several genes encoding enzymes involved in plant interaction, such as cellulases, suggests adaptation of the fungus to its native forest environment. Although the overall genetic profile of this strain aligns with the broad‐spectrum activity typical of *B. bassiana*, the observed sequence variability highlights its uniqueness and may underlie distinctive biological traits that warrant further investigation.^46^

According to the results of this study, *B. bassiana* strain UNISS22 appears well‐adapted to oak plants and shows significant potential for the management of *Coraebus* borers populations, particularly because of the endophagous habits of the larvae. Beyond targeting larvae, the observed susceptibility of adults presents additional management opportunities. For instance, infecting adults in the field via inoculation devices could enable them to serve as vectors, spreading the fungus to other insects or to plants where immature stages develop.^47^, ^48^ Notably, the aptitude of *Coraebus* adults to carry fungi adhered to their bodies is well‐documented.^4^

However, successful field application will require the development of effective techniques to target the damaging larvae and achieve the desired level of efficacy.^49^ Long‐term studies and continuous monitoring are essential to evaluate not only the efficacy, but also the safety of *B. bassiana* strain UNISS22 to non‐target species, as demonstrated in similar assessments conducted with other *B. bassiana* strains,^50^ to ensure its suitability for integrated pest management programmes targeting *Coraebus* species.

Regarding the challenges of managing *Coraebus* infestations in forests, the availability of a well‐characterized strain of *B. bassiana* could complement existing control measures, including natural enemies, adult trapping and removal of infested branches or plants. Thus, the results of this study contribute to the development of eco‐friendly integrated management strategies aimed at protecting forests from *Coraebus* beetles.

## CONFLICT OF INTEREST

The authors declare that there is no conflict of interest.

## Supporting information


**Table S1.** Comparative Sequence Analysis of Selected Genes Related to Endophytism, Pathogenesis, and Virulence in *Beauveria bassiana* strains UNISS22 and ATCC74040.

## Data Availability

Sequences of genes analysed in this work are available on the NCBI public repository. Other data that support the findings of this study are available upon reasonable request.
