# Supplementary material for: Potential of endophytic Beauveria bassiana against Coraebus (Coleoptera: Buprestidae) oak borers
Source: Pest Manag Sci. 2025 Dec 30;82(4):3523–31. doi: 10.1002/ps.70473 (PMC12976180; doi:10.1002/ps.70473)
Supplement: Supplementary file 1 — Table S1. Comparative Sequence Analysis of Selected Genes Related to Endophytism, Pathogenesis, and Virulence in Beauveria bassiana strains UNISS22 and ATCC74040. [file PS-82-3523-s001.pdf]

Supplementary Table (S1) - Comparative Sequence Analysis of Selected Genes Related to Endophytism, Pathogenesis, and Virulence in *Beauveria bassiana* strains UNISS22 and ATCC74040

qseqid	sseqid	pident	length	mismatch	gapopen	qstart	qend	sstart	send	evalue	bitscore
hydrophobin-like_protein_UNISS22	JABAOI010000004.1	87.984	258	28	1	1	255	58004	57747	9.48e-82	302
Hydrophobin-2_UNISS22	JABAOI010000713.1	79.936	314	43	11	5	299	8260	8572	4.87e-55	213
Hydrophobin-2_UNISS22	JABAOI010000713.1	78.964	309	48	9	1	302	7854	8152	1.76e-49	195
Hydrophobin-2_UNISS22	JABAOI010000713.1	89.231	65	7	0	204	268	8405	8469	1.43e-15	82.4
adhesin-protein_Mad1_UNISS22	JABAOI010000855.1	86.836	1223	102	21	572	1772	4263	3078	0.0	1312
adhesin-protein_Mad1_UNISS22	JABAOI010000855.1	89.676	678	50	7	1	677	5482	4824	0.0	846
adhesin-protein_Mad1_UNISS22	JABAOI010000855.1	90.385	52	5	0	718	769	4186	4135	6.98e-11	69.4
chitinase_18-3_UNISS22	JABAOI010000263.1	84.211	1159	82	40	1	1083	8564	7431	0.0	1033
chitinase_18-4_UNISS22	JABAOI010000498.1	96.416	586	21	0	317	902	4209	4794	0.0	966
chitinase_18-4_UNISS22	JABAOI010000498.1	94.953	317	16	0	1	317	3849	4165	3.77e-140	497
subtilase-like_protein_UNISS22	JABAOI010000686.1	91.520	625	44	2	1	625	7506	8121	0.0	852
subtilase-like_protein_UNISS22	JABAOI010000686.1	93.069	303	21	0	625	927	8174	8476	7.15e-124	444
subtilase-like_protein_UNISS22	JABAOI010000686.1	91.327	196	17	0	928	1123	8536	8731	4.62e-71	268
subtilase-like_protein_UNISS22	JABAOI010000686.1	88.889	162	18	0	1123	1284	8797	8958	1.71e-50	200
cellulase-like-protein_UNISS22	JABAOI010000061.1	91.236	1278	85	15	1	1251	73021	71744	0.0	1714
cellulase_UNISS22	JABAOI010000211.1	92.906	1029	73	0	466	1494	29648	28620	0.0	1496
cellulase_UNISS22	JABAOI010000211.1	94.624	465	25	0	1	465	30169	29705	0.0	721
aspartokinase-like-protein_UNISS22	JABAOI010000073.1	87.556	675	44	9	336	970	61391	62065	0.0	745
aspartokinase-like-protein_UNISS22	JABAOI010000073.1	92.901	324	23	0	1	324	61016	61339	3.04e-132	472
Cytochrome_P450_CYP65T7_UNISS22	JABAOI010000140.1	93.461	1147	73	2	1	1146	23895	22750	0.0	1701
Cytochrome_P450_CYP65T7_UNISS22	JABAOI010000140.1	91.944	360	26	2	1138	1494	22679	22320	4.88e-141	501
Cytochrome_P450_CYP65A1_UNISS22	JABAOI010000140.1	94.133	784	46	0	1	784	21588	20805	0.0	1194
beta-1,6-glucanase_precursor_UNISS22	JABAOI010000330.1	93.794	1128	67	1	175	1299	2256	1129	0.0	1692
beta-1,6-glucanase_precursor_UNISS22	JABAOI010000330.1	94.318	176	10	0	1	176	2486	2311	1.30e-71	270
alkaline_serine_protease_AorO_UNISS22	JABAOI010000946.1	94.078	743	44	0	1170	1912	7718	6976	0.0	1129
alkaline_serine_protease_AorO_UNISS22	JABAOI010000946.1	93.491	507	33	0	663	1169	8284	7778	0.0	754
alkaline_serine_protease_AorO_UNISS22	JABAOI010000484.1	93.900	459	28	0	207	665	21720	22178	0.0	693
alkaline_serine_protease_AorO_UNISS22	JABAOI010000484.1	97.674	215	5	0	1	215	21430	21644	1.85e-101	370
glucan-1,3-beta-glucosidase_UNISS22	JABAOI010000009.1	89.631	1408	111	13	1	1401	98186	96807	0.0	1759
subtilisin-like_protease_PR1G_UNISS22	JABAOI010000063.1	92.287	726	56	0	472	1197	42762	43487	0.0	1031
subtilisin-like_protease_PR1G_UNISS22	JABAOI010000063.1	88.742	302	26	6	1	296	42006	42305	1.92e-99	363
subtilisin-like_protease_PR1G_UNISS22	JABAOI010000063.1	96.364	55	2	0	418	472	42595	42649	9.99e-18	91.6
beauvericin_BBA_09727_UNISS22	JABAOI010000349.1	90.092	9376	834	40	1	9297	11390	2031	0.0	12080
endoglucanase_UNISS22	JABAOI010000417.1	95.740	446	19	0	245	690	19987	20432	0.0	719
endoglucanase_UNISS22	JABAOI010000417.1	95.918	245	10	0	1	245	20498	20742	2.99e-110	398
tenellin_polyketide_synthase_UNISS22	JABAOI010000044.1	86.318	7141	810	69	4485	11480	56229	63347	0.0	7620
tenellin_polyketide_synthase_UNISS22	JABAOI010000044.1	87.833	3230	375	11	1270	4484	52906	56132	0.0	3771
tenellin_polyketide_synthase_UNISS22	JABAOI010000044.1	90.610	1278	111	6	1	1269	51516	52793	0.0	1687
Cytochrome_P450_CYP623C1_UNISS22	JABAOI010000044.1	88.198	1110	127	4	1	1108	48763	47656	0.0	1321
beta-glucosidase-6_UNISS22	JABAOI010000447.1	95.356	1701	79	0	1	1701	5979	4279	0.0	2704

Comparative sequence analyses were conducted using the BLAST+ v2.16.0 suite of NCBI (Camacho et al., 2009).
